# Investigation of the Antioxidant Activity of Hydroxycinnamic Acids, Hydroxybenzoic Acids, and Their Synthetic Diazomethane Derivatives

**DOI:** 10.3390/molecules31091375

**Published:** 2026-04-22

**Authors:** Katherine Liset Ortiz Paternina, Michel Murillo Acosta, Joaquín Hernández Fernández

**Affiliations:** 1 Chemistry Program, Department of Natural and Exact Sciences, San Pablo Campus, Universidad de Cartagena, Cartagena de Indias D.T. y C., Cartagena 130015, Colombia; 2 Department of Civil and Environmental, Universidad de la Costa, Barranquilla 080002, Colombia; mmurillo4@cuc.edu.co; 3 Department of Natural and Exact Science, Universidad de la Costa, Barranquilla 080002, Colombia; 4 Grupo de Investigación GIA, Fundacion Universitaria Tecnologico Comfenalco, Cr 44 D N 30A, 91, Cartagena 130015, Colombia

**Keywords:** phenolic extracts, diazomethane methylation, antioxidant activity, FRAP assay, iron chelation (Ca–Fe), density functional theory (DFT)

## Abstract

Phenolic-rich extracts from *Satureja montana* were evaluated before and after diazomethane treatment to determine how chemical derivatization influences their antioxidant capacity. Native and modified extracts were compared experimentally by measuring total phenolic content, ferric reducing antioxidant power (FRAP), and Fe^2+^-chelating ability. EN1 exhibited the highest concentration of phenolic compounds, reaching 1278.54 mmol/g, whereas EM2 retained only 1.99 mmol/g. In the FRAP assay, reducing power followed the order EN1 (9.36) > EN2 (3.72) > EM2 (2.08), with EM2 still exceeding caffeic, chlorogenic, and ferulic acids. In contrast, the modified extracts showed superior metal chelating capacity, with EM1 and EM2 displaying IC_50_ values of 0.70 and 0.82 mg/mL, respectively, both markedly lower than those of the native extracts and the pure standards. To rationalize these differences, a DFT study was performed at the B3LYP/6-311++G(d,p) level, examining 18 proposed phenolic acids and their methylated derivatives associated with the extracts. All methylation reactions were thermodynamically favorable, particularly for compounds **18** (−57.10 kcal/mol), **16** (−53.96), **6** (−53.34), and **3**, **9**, and **11** (−52.71). Solvent effects were found to be structure-dependent: caffeic acid showed BDE values of 72.29, 73.59, and 74.43 kcal/mol in the gas phase, water, and benzene, respectively, whereas syringic acid displayed values of 80.44, 77.09, and 80.65 kcal/mol under the same conditions. Likewise, the ionization potential of caffeic acid decreased from 180.09 kcal/mol in the gas phase to 133.26 kcal/mol in water and 154.22 kcal/mol in benzene. Among all analyzed species, methyl 3,4-dihydroxycinnamate exhibited the lowest BDE (71.60 kcal/mol) as well as the most favorable ΔG°r toward HOO• (−11.06 kcal/mol).

## 1. Introduction

Plant-derived phenolic compounds have attracted considerable interest because of their role as natural antioxidants and the remarkable structural diversity found in extracts from aromatic and medicinal species [1]. The antioxidant function of these compounds is closely associated with their ability to neutralize free radicals, donate hydrogen atoms or electrons, and stabilize the resulting radical species through resonance effects and electronic delocalization [2,3]. Accordingly, the antioxidant reactivity of these molecules depends not only on their presence in the extract, but also on specific structural features, including the number and position of hydroxyl groups, the presence of methoxy substituents, the degree of conjugation, and the properties of the carboxylic substituent [4,5].

Phenolic acids, particularly hydroxycinnamic and hydroxybenzoic acid derivatives, are among the most important phenolic metabolites in the plant kingdom. Caffeic, ferulic, gallic, syringic, chlorogenic, and rosmarinic acids are widely recognized for their notable antioxidant properties and are found in a broad range of plant species [6,7]. The structural features of these compounds facilitate the inactivation of reactive species; however, this capacity can vary substantially in response to subtle changes in their functional groups. Therefore, examining structural modifications is essential for identifying which changes enhance or impair their antioxidant effectiveness [2].

In this context, *Satureja montana* stands out for its strong aroma and its high levels of phenolic metabolites, which are recognized for their bioactive properties [8]. Because its extracts contain phenolic acids, this plant matrix provides an excellent model for examining how chemical composition influences the overall antioxidant response [9,10]. However, the activity observed in an extract should not be attributed solely to total phenolic content; rather, it should be understood as the result of the combined contributions of multiple molecules, each with distinct reducing, metal chelating, or radical scavenging capacities [11,12,13]. This complexity underscores the need to investigate not only the native extracts, but also the chemical and functional changes that arise from controlled modifications of their components.

Modifying the structure of natural antioxidants is an effective way to gain deeper insight into structure–activity relationships and to identify which molecular changes have the greatest impact on their reactivity [14,15,16]. Diazomethane methylation is one such transformation of particular interest to chemists, because it enables the conversion of carboxylic groups into methyl esters and, depending on the system, may also affect other oxygen-containing functional groups [17]. This type of modification can alter molecular polarity, charge distribution, the stability of the radicals formed after hydrogen abstraction, and, more generally, the thermochemical factors that govern antioxidant efficiency [18,19]. Accordingly, its relevance lies not only in the generation of methylated derivatives, but also in the opportunity it provides to evaluate, with precision, how methylation modifies the antioxidant behavior of the compounds present in the extract, either by favoring or impairing pathways such as hydrogen donation, electron transfer, or metal chelation.

From a mechanistic perspective, this type of investigation requires tools capable of correlating the molecular structure with the experimental response. In this regard, density functional theory (DFT) has become a particularly suitable approach for studying phenolic compounds, as it allows the estimation of electronic and thermochemical properties that are directly relevant to antioxidant activity, including bond dissociation energy (BDE) [20,21,22], ionization potential (IP), frontier molecular orbital energies, and electrostatic potential distribution [23,24,25]. Unlike an exclusively experimental approach, computational analysis makes it possible to compare structurally related molecules, evaluate how the medium affects their reactivity, and examine the interactions among different reactive regions within the same molecule. As a result, DFT not only complements experimental data, but also helps explain why some compounds exhibit greater antioxidant potency than others.

From this perspective, it is scientifically important to investigate native phenolic extracts together with their derivatization products in an integrated manner. Rather than assuming that chemical modification inherently enhances antioxidant activity, it is essential to examine how diazomethane methylation affects the balance among different measurable antioxidant responses, such as the reducing power and metal chelating capacity. This approach is particularly relevant in complex natural systems, where a structural alteration may diminish certain chemical functions while simultaneously enhancing others, thereby giving rise to a distinct and potentially heterogeneous antioxidant profile.

This study aimed to examine *Satureja montana* extracts before and after diazomethane modification in order to evaluate the effects of this chemical derivatization on their phenolic content and antioxidant properties. To this end, native and modified extracts were comparatively analyzed through total phenolic content determination, the FRAP assay, and iron-chelating capacity measurements. In parallel, a DFT-based computational analysis was performed on selected phenolic acids and their methylated derivatives to establish a relationship between the structural changes induced by methylation and the electronic and thermochemical parameters associated with antioxidant reactivity. Accordingly, this work seeks to correlate extract composition and diazomethane-induced molecular changes with the antioxidant response observed from both experimental and theoretical perspectives.

## 2. Results

### 2.1. Phenolic Profile of Native and Diazomethane-Modified Extracts

**Figure 1:**
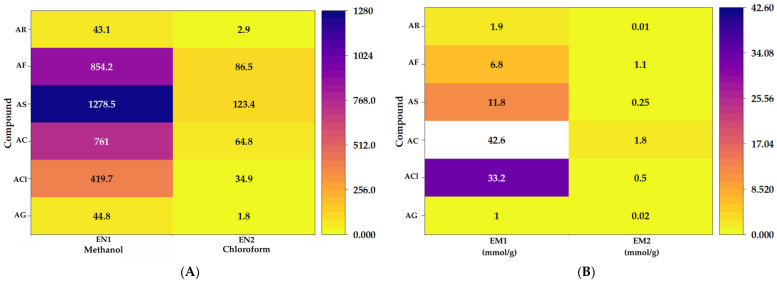
Total phenolic content of native and diazomethane-modified *Satureja montana* extracts. EN1 and EN2 correspond to the unmodified methanolic and chloroform-derived extracts, respectively (**A**), whereas EM1 and EM2 correspond to the diazomethane-treated fractions (**B**).

The first clear effect of the diazomethane treatment was a big change in the phenolic composition of the extracts. The unaltered samples had a lot of differences in their composition. The total phenolic content was highest in EN1 (1278.54 mmol/g) and lowest in EN2 (132.17 mmol/g). After chemical treatment, the amount of phenolic left over dropped a lot. EM1 still had 42.60 mmol/g left, but EM2 only had 1.99 mmol/g left. From a chemical point of view, this behavior is important because it shows that the derivatization process affected different extract fractions in different ways (Figure 1A,B). Consequently, it is infeasible to consider the two altered extracts as compositionally identical systems. The high residual value of EM1 indicates either a partial recovery of redox-active species during the workup process or incomplete derivatization. In contrast, the significant decrease seen in EM2 corresponds to a more complete chemical transformation of the oxygenated functionalities that affect the Folin–Ciocalteu response.

This distinction is significant because, in plant extracts, the total phenolic content does not merely indicate quantity, but rather the existence of functional groups that can reduce the Folin reagent under the specified assay conditions. Consequently, the significant reduction in the signal following diazomethane treatment should not be regarded solely as a decrease in concentration; instead, it should be seen as an indication that the chemical environment of the oxygenated groups was significantly modified. From a mechanistic perspective, this aligns seamlessly with a derivatization process that alters the reactivity of phenolic acids and associated components without the requisite removal of all oxygen donor sites from the extract matrix. That difference is very important when we look at the antioxidant tests in the next section.

During the extraction phase, it was clear that the solvent had an effect. The analysis of the identified phenolic acids indicated that the methanolic fraction displayed higher concentrations than the chloroform fraction, implying that the solvent’s polarity substantially affected the extraction of the oxygenated phenolic compounds. This behavior is consistent with the high number of hydroxylated and carboxylated structures in the compounds being studied. These structures are more stable and easier to extract in a polar protic medium like methanol than in a less polar solvent like chloroform. The similarity in the qualitative composition of both extracts, along with the consistently higher levels in methanol, suggests that the solvent’s main effect was on the amount of extraction rather than the type of extraction, improving extraction efficiency without changing the phenolic profile much.

### 2.2. Antioxidant Activity of Native and Modified Extracts

**Figure 2:**
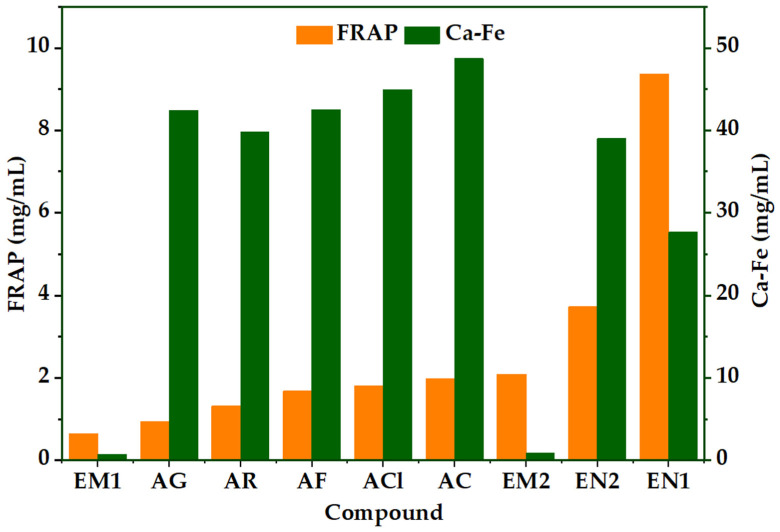
Evaluation of the antioxidant performance of native extracts, diazomethane-modified extracts, and pure phenolic standards using the iron reducing antioxidant power (FRAP) and Fe^2+^ chelating activity (Ca–Fe assay).

The antioxidant assays indicate that diazomethane treatment results in a functional redistribution of activity rather than a straightforward gain or loss. The native extracts had the strongest reducing response in the FRAP assay, in the order of EN1 (9.36) > EN2 (3.72) > EM2 (2.08) > AC (1.97) > ACl (1.79) > AF (1.67) > AR > AG > EM1. So, even though EM2 was still more reducing than some pure standards, both modified extracts were not as good as the native extracts at reducing ferric ions (Figure 2). Since FRAP mainly tests how well a substance can donate electrons under the conditions of the assay, this trend is chemically consistent with a decrease in the number or availability of free hydrogen- and electron-donating phenolic groups after methylation. In other words, native extracts keep a larger pool of directly redox-active functionalities, while chemical derivatization makes that pool smaller.

The behavior changes a lot in the Ca–Fe test. The modified extracts were clearly better, with IC_50_ values of 0.70 mg/mL for EM1 and 0.82 mg/mL for EM2. In contrast, the IC_50_ values for EN1 and EN2 were 27.61 and 39.01 mg/mL, respectively, and the IC_50_ values for the pure phenolic standards were much higher. This change in relation to FRAP is important from a chemical point of view. If the same chemical change had made antioxidant capacity stronger across the board, both tests should have gotten better in the same way. The data, on the other hand, show that diazomethane treatment weakens the overall reducing response while also promoting Fe^2+^ sequestration. The most logical explanation is that methylation changed the balance of oxygen-containing donor sites and the relative contribution of species in the extract. This made the modified matrices less effective as global reductants but more competitive as Fe^2+^-binding systems. This does not establish the identity of the specific chelating species in EM1 and EM2; however, it does indicate that the chemical modification altered the functional antioxidant mechanism of the extract.

### 2.3. Thermodynamic Feasibility of Diazomethane Methylation

**Figure 3:**
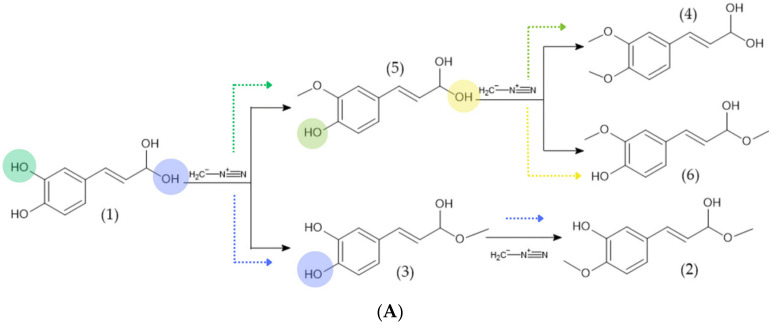

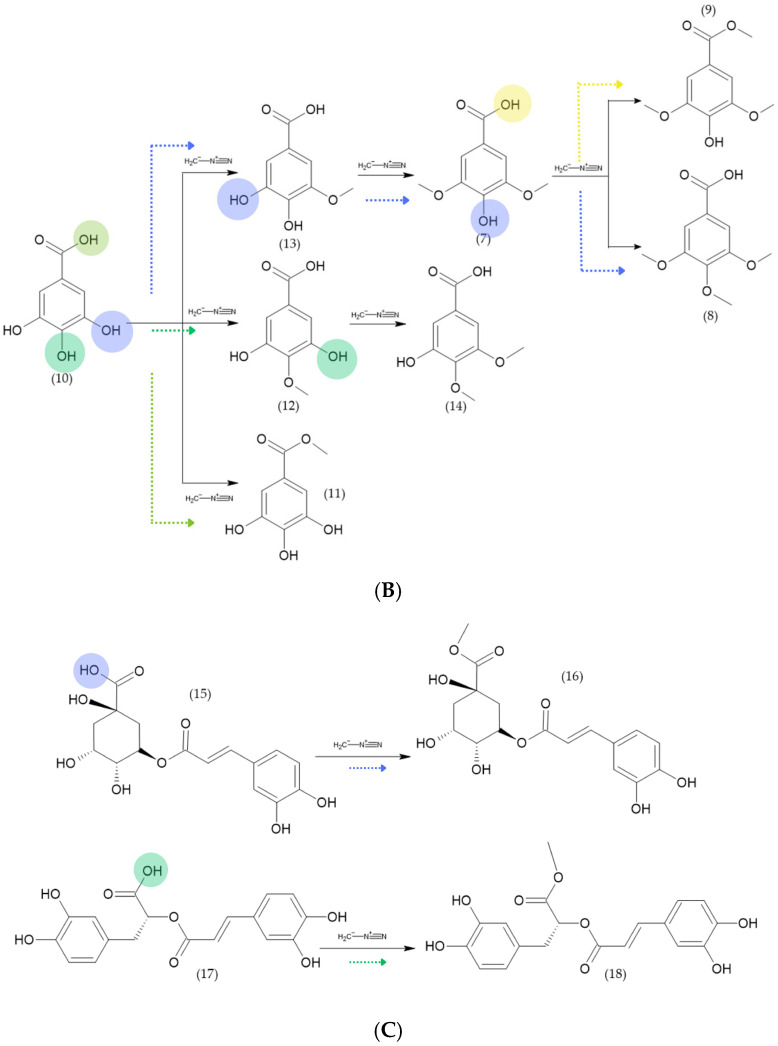
Reaction between diazomethane and natural antioxidants; (**A**) possible steps to obtain derivatives of caffeic and ferulic acids; (**B**) possible steps to obtain derivatives of gallic and syringic acids; (**C**) possible steps to obtain derivatives of chlorogenic and rosmarinic acids. Colored circles indicate the specific hydroxyl or carboxylic sites considered for methylation in each precursor, whereas dashed arrows of the same color identify the corresponding methylation pathway leading to each proposed derivative.

The plausibility of the chemical changes inferred from the experimental assays at the molecular level was assessed by evaluating the methylation of representative phenolic acids with diazomethane using DFT (Figure 3). All computed methylation reactions were exergonic, demonstrating that the synthesis of the suggested derivatives is thermodynamically viable. The most favorable products were compounds **18** (−57.10 kcal/mol), **16** (−53.96 kcal/mol), **6** (−53.34 kcal/mol), and compounds **3**, **9**, and **11** (−52.71 kcal/mol). These values are not just random numbers; they show that diazomethane treatment can chemically make a group of methylated derivatives under the conditions we studied.

The thermodynamic findings indicate that diazomethane-mediated methylation is chemically feasible for the representative phenolic acids associated with the extracts, providing a solid molecular basis for the compositional changes proposed by the experimental data. Within this framework, the calculated reaction free energies do not endeavor to ascertain the exact composition of EM1 and EM2; rather, they strive to outline the spectrum of energetically viable methylated derivatives that could arise under the designated reaction conditions. The computational analysis supports the interpretation that the notable decrease in residual phenolic content following treatment, particularly in EM2, corresponds with the formation of thermodynamically favorable methylation products derived from the native phenolic components.

The synthesis of compound **3** from caffeic acid within this collection is particularly significant, as this derivative subsequently stands out as one of the most promising contenders for hydrogen donation. Consequently, the thermodynamics of methylation are intrinsically linked to the discourse on antioxidants, as they reveal chemically accessible products whose electronic and thermodynamic characteristics diverge from those of the parent acids. A comparable analysis is relevant for compounds **16** and **18**, which are derived from chlorogenic and rosmarinic acids, respectively. Their advantageous formation energies suggest that more extensive multifunctional phenolic systems are likewise prone to structural reorganization induced by diazomethane.

### 2.4. Global Reactivity Descriptors and Frontier Molecular Orbitals

A frontier molecular orbital analysis provides a more rigorous framework for interpreting the electronic factors that control the antioxidant behavior of phenolic systems. The HOMO and LUMO energies are particularly informative because they define the ease of charge redistribution within the molecule and, therefore, its ability to participate in electron-transfer and radical-quenching processes. In general, a smaller HOMO–LUMO gap is associated with greater electronic softness and a higher capacity for polarization, both of which favor chemical reactivity by facilitating intramolecular charge displacement [26]. Conversely, larger gaps usually reflect electronically more rigid systems with lower responsiveness. Within this framework, the HOMO energy is especially relevant for antioxidant compounds because it is directly connected to electron-donating ability: a higher HOMO energy indicates that electron removal is energetically more accessible, whereas a lower HOMO energy suggests a less favorable donor character. For phenolic antioxidants, the spatial localization of the HOMO is equally important, since it identifies the regions of highest electronic availability and therefore the sites most likely to participate in hydrogen abstraction or electron-transfer events [27]. In molecules bearing multiple hydroxyl substituents, the frontier electron density is preferentially distributed over the oxygenated aromatic region, which is consistent with the formation of resonance-stabilized phenoxyl radicals after hydrogen loss. This behavior also agrees with low spin-density localization at the resulting O• centers, indicating that radical stabilization is governed not only by the presence of donor groups, but also by the extent of electronic delocalization across the molecular scaffold [9].

**Figure 4:**
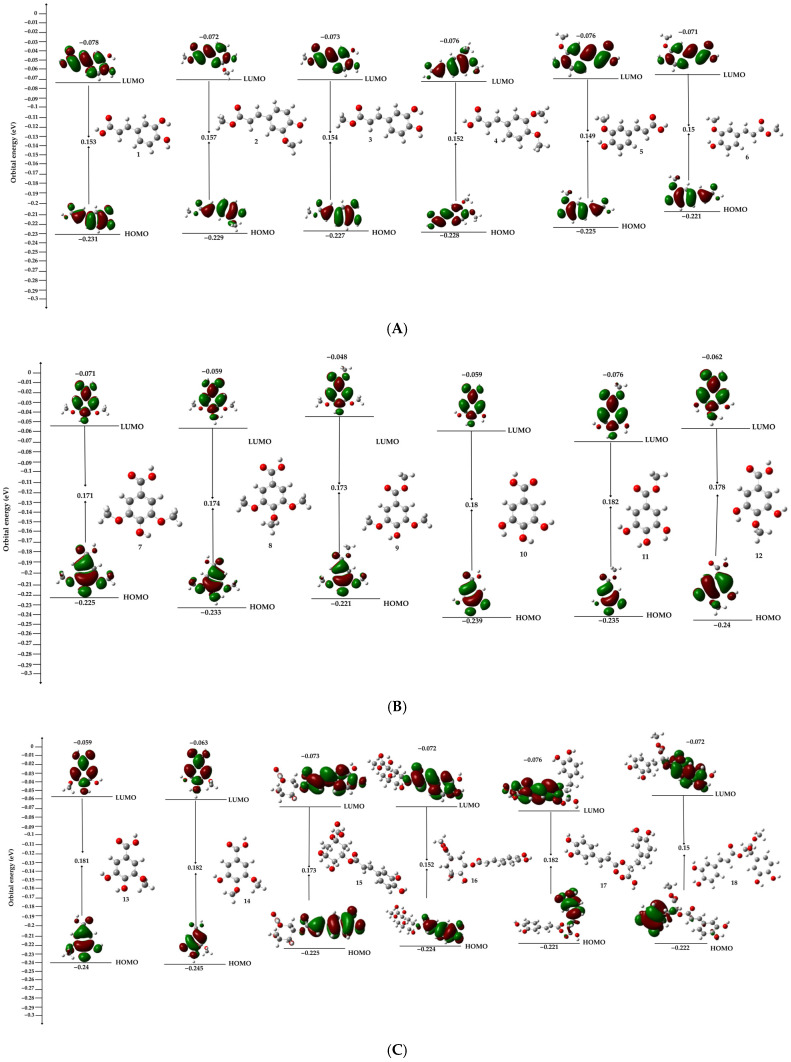
Frontier molecular orbitals (HOMO and LUMO) and corresponding HOMO–LUMO energy gaps of compounds **1**–**18**, organized as follows: (**A**) compounds **1**–**6**, (**B**) compounds **7**–**12**, and (**C**) compounds **13**–**18**.

The analysis of frontier-orbital and global descriptors categorizes the examined species into two principal electronic regimes. Hydroxycinnamic acids, along with chlorogenic and rosmarinic scaffolds and their methyl esters, are situated in the lower-gap region of the series (ΔE = 0.145–0.157 eV), while the hydroxybenzoic derivatives consistently exhibit larger gaps (0.171–0.182 eV) (Figure 4). This distinction illustrates the enhanced π-delocalization of the former group, wherein the aromatic ring maintains electronic coupling with an unsaturated or extended oxygenated side chain, thereby promoting charge redistribution and reducing the energetic expense associated with electronic reorganization. In contrast, the benzoic derivatives exhibit characteristics of greater electronic hardness, displaying reduced softness and a more constrained delocalization. Consequently, the antioxidant properties within this group cannot be understood merely by counting hydroxyl groups; instead, they should be assessed based on the molecular structure’s capacity to stabilize charge redistribution following oxidation or the loss of hydrogen.

This trend is preserved upon comparison of structurally related pairs. Esterification of the carboxylic group produces only minor changes in ΔE, as observed for caffeic acid/methyl 3,4-dihydroxycinnamate, ferulic acid/ferulic acid methyl ester, and the chlorogenic and rosmarinic pairs, indicating that methylation does not disrupt the main conjugated donor framework (Table 1). Instead, it subtly modifies the HOMO energy, dipole moment, and polarizability, thereby altering charge distribution without suppressing electronic responsiveness. The HOMO remains predominantly localized over the oxygenated aromatic region and, in the most conjugated systems, also extends toward the side-chain fragment, which is consistent with these regions acting as the principal sites for electron donation and radical stabilization. Accordingly, the lowest hardness and highest softness values are concentrated in the more conjugated and multifunctional structures, confirming that antioxidant reactivity in this series is governed primarily by conjugation-assisted electronic delocalization rather than by oxygen content alone.

**Figure 5:**
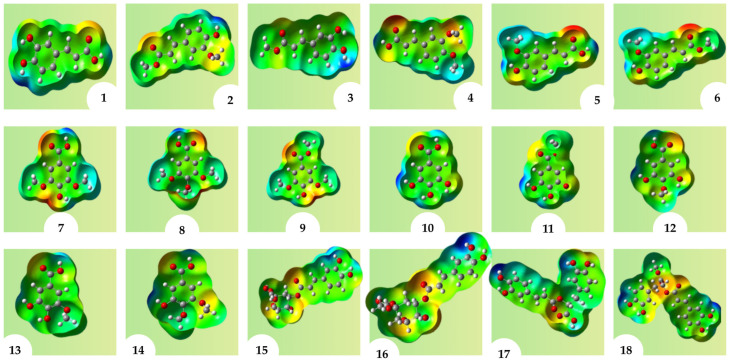
Molecular electrostatic potential map of compounds **1**–**18**. The surface color scale ranges from red to blue, where red denotes the most electron-rich/negative potential regions, blue denotes the most electron-deficient/positive potential regions, and green to yellow indicates intermediate electrostatic potential values. The spheres represent the atoms, with gray for carbon (C), red for oxygen (O), and white for hydrogen (H).

The MEP serves as a crucial descriptor in validating various aspects of molecular responsiveness. The 3-D surface of MEP offers insights into the spatial distribution, morphology, magnitude of positive and negative charges, and overall electrostatic potentials of molecules. This dataset elucidates the structural correlations and responsiveness concerning electrophilic and nucleophilic interactions. As depicted in Figure 5, regions with the maximum negative electronic potential (highlighted in red) indicate preferred sites for electrophilic attack. Conversely, molecules bearing radical charges exhibit an affinity towards regions of positive electrostatic potential (depicted in blue).

### 2.5. Solvent Effects on Bond Dissociation Energy and Ionization Potential

**Figure 6:**
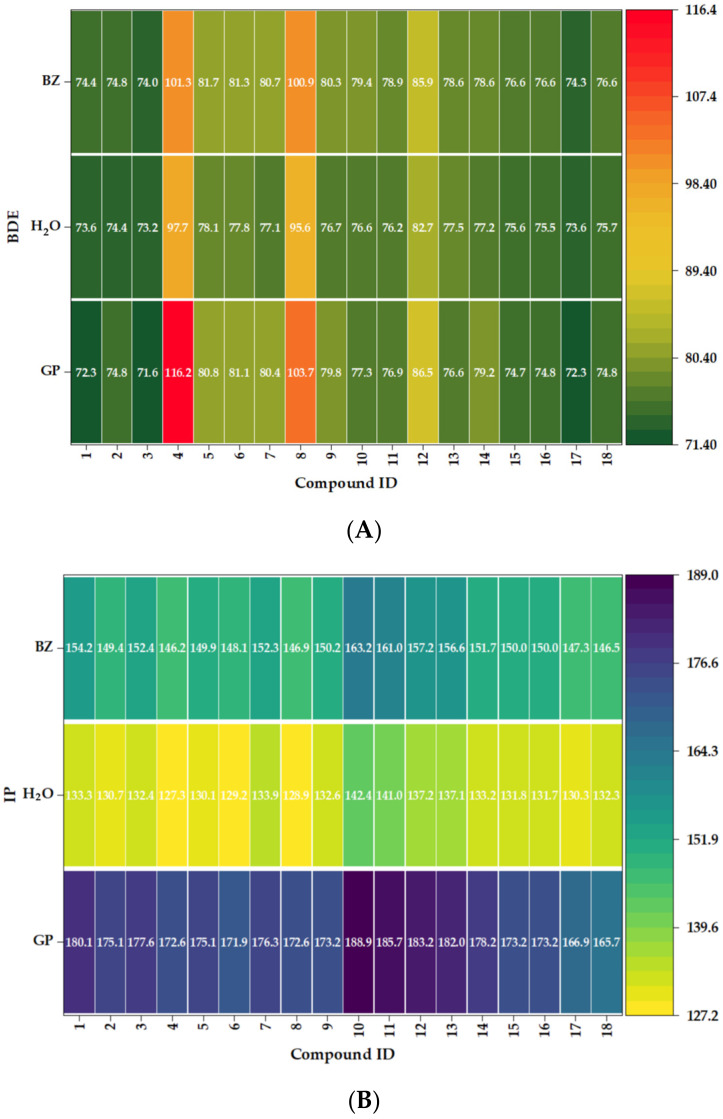
Heat map representation of bond dissociation energy (BDE) and ionization potential (IP) values for compounds **1**–**18** in the gas phase (GP), water (H_2_O), and benzene (BZ): (**A**) BDE and (**B**) IP.

The bond dissociation energy (BDE) and ionization potential (IP) values obtained in the gas phase, water, and benzene were arranged into heat maps to shed light on the solvent dependency of antioxidant reactivity (Figure 6A,B). This example shows that the medium’s effect is not consistent across all chemicals, but rather varies across them. In the BDE map (Figure 6A), certain compounds, such as caffeic acid, exhibit a consistent increase from the gas phase to water and benzene, with values of 72.29, 73.59, and 74.43 kcal/mol, respectively. This indicates that the likelihood of breaking the O–H bond diminishes in condensed media. Syringic acid exhibits a lower bond dissociation energy in aqueous solution (80.44 kcal/mol) compared to its value in the gas phase (77.09 kcal/mol). Benzene, conversely, exhibits a value that closely aligns with the gas-phase result (80.65 kcal/mol). Methyl gallate and methyl syringate exhibit comparable reductions when solubilized in a solvent. The results suggest that the capacity for hydrogen donation is shaped not by a singular trend in solvents, but rather by the interplay of substitution patterns, conjugation, and the differential stabilization of radical species.

In all compounds, ionization potential values notably decrease in solution, especially in water, indicating that the donation of electrons becomes energetically more favorable in polar environments (Figure 6B). For example, the energy of caffeic acid transitions from 180.09 kcal/mol in the gas phase to 133.26 kcal/mol in water and 154.22 kcal/mol in benzene. The energy alterations of rosmarinic acid are observed to shift from 166.92 to 130.28 and subsequently to 147.30 kcal/mol, respectively. The solvent influences both the hydrogen donation via bond dissociation energy and the electron transfer through ionization potential. The heat maps illustrate that the reactivity of antioxidants in this series is influenced by the molecular structure as well as the characteristics of the surrounding medium.

### 2.6. Thermodynamics of Hydrogen Atom Transfer Toward the Hydroperoxyl Radical

**Figure 7:**
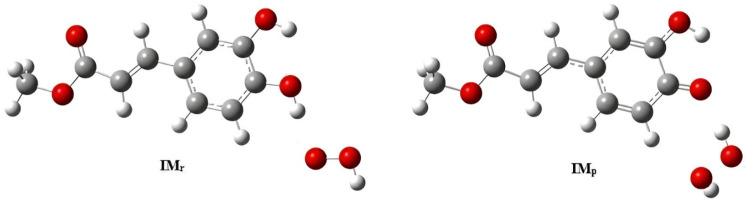
Pre-reactive complex (IMr) and product complex (IMp) are involved in the reaction of methyl 3,4-dihydroxycinnamate with the hydroperoxyl radical (HOO•).

The HAT analysis of the hydroperoxyl radical is a precise thermodynamic test of the donor capacity that BDE predicts (Figure 7).

Methyl 3,4-dihydroxycinnamate has the best reaction free energy of the structures that were tested, with ΔG°r = −11.06 kcal/mol. Caffeic acid is a close second, with −10.47 kcal/mol. Gallic acid is still good (−8.02 kcal/mol), while syringic acid and chlorogenic acid are not as good, with values of −4.78 and −2.25 kcal/mol, respectively (Table 2). This arrangement is chemically compatible with the bottom BDE area that the derivatives with catechol and strong conjugation occupy. In these systems, removing hydrogen creates radicals that are more stable because of resonance and charge delocalization with the help of substituents. This lowers the total thermodynamic cost of the HAT process.

**Figure 8:**
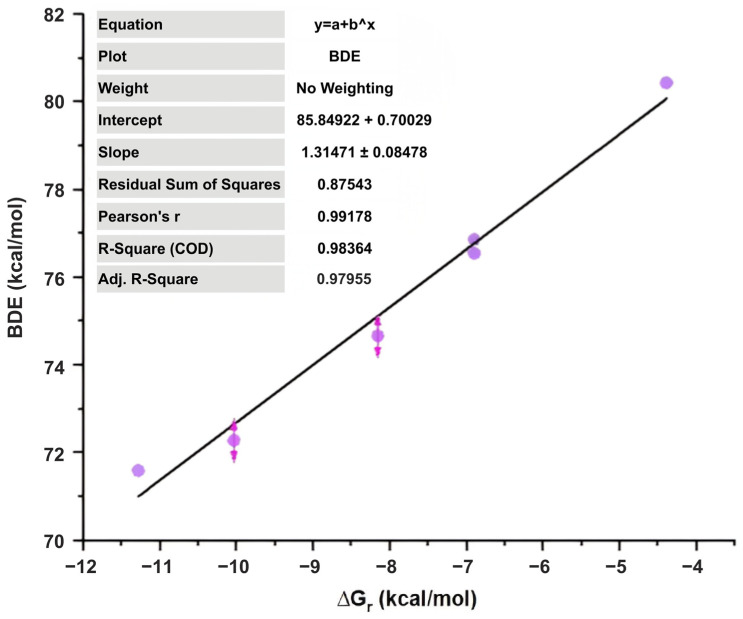
Correlation between bond dissociation energy (BDE) and reaction Gibbs free energy (ΔGᵣ) for hydrogen atom transfer to the hydroperoxyl radical (HOO•).

The correlation (r = 0.992) between BDE and ΔGᵣ for HOO• reactions reveals that the thermodynamic expenditure of cleaving the O-H bond is strongly related to the favorability of hydrogen atom migration within the investigated set (Figure 8). As a result, molecules with lower bond dissociation energy values have significantly higher negative reaction free energies, confirming the hypothesis that hydrogen donation is a thermodynamically favored pathway for the most reactive structures. The results present a unified molecular structure in which the promotion of bond dissociation is closely associated with the motivation for hydroperoxyl radical scavenging.

In this context, chemical 3 is very important since it ties the methylation analysis to the antioxidant mechanism. The production of this compound is thermodynamically beneficial in the diazomethane derivatization scheme, but it also has the lowest BDE and the most favorable ΔGᵣ value in the HOO• model reaction. This combination suggests compound **3** as the most likely choice for understanding how derivatization might produce individual species with better hydrogen-donating thermodynamic properties. At the same time, this behavior should not be applied directly to the global extract response. The antioxidant performance of the extract is determined by the combined contribution of multiple constituents, their relative abundance, and the coexistence of different reactive functions, so the most advantageous single-molecule donor does not always determine the overall experimental response of the multicomponent system.

### 2.7. Theoretical–Experimental Agreement for the Pure Phenolic Standards

**Figure 9:**
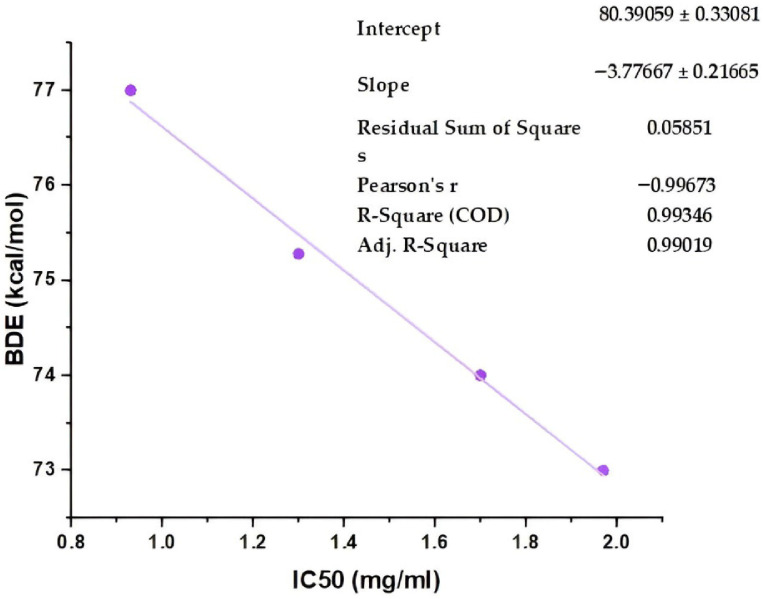
Relationship between bond dissociation energy (BDE) and experimental FRAP response for the pure phenolic standards.

To strengthen the validity of the theoretical findings, a comparison was made between the theoretical BDE of each antioxidant in its pure form and the experimentally determined IC_50_ value obtained by the FRAP assay. This comparison revealed a strong, direct relationship between the theoretical BDE and the IC_50_ (r = 0.996, *p* = 0.0032 < 0.05). This outcome underscores the credibility of the theoretical computations, affirming the reliability and consistency of the employed framework in forecasting the antioxidant potency of the analyzed compounds (Figure 9).

## 3. Discussion

The electronic descriptors indicate a clear distinction between two structural domains in the analyzed series. Hydroxycinnamic acids, chlorogenic and rosmarinic scaffolds, together with their methyl esters, occupy the lower-gap region of the dataset. In contrast, hydroxybenzoic derivatives are progressively shifted towards wider HOMO-LUMO separations. This difference is important from a chemical point of view because it shows that the first group has a higher level of π-delocalization. In this group, the aromatic ring stays electrically attached to an unsaturated or extended oxygenated fragment, which lets charge move about after oxidation. Benzoic derivatives, on the other hand, act like systems that are harder to electrify, with less softness and less delocalization. Thus, antioxidant behavior in this set is governed less by the absolute number of oxygenated substituents than by the molecular scaffold’s ability to stabilize charge redistribution after electron transfer or hydrogen loss, which is consistent with previous DFT studies that identified conjugation and catechol-type substitution as important factors in antioxidant reactivity, particularly in the analyses reported by Bendaas et al. and Ma and Wang [28,29].

This pattern is maintained in the structurally analogous methylated derivatives. Esterification of the carboxylic group results in negligible alterations to the HOMO–LUMO gap for the caffeic, ferulic, chlorogenic, and rosmarinic pairs, suggesting that methylation does not inhibit the primary conjugated donor framework but rather restructures charge distribution and molecular flexibility. This behavior aligns with previous computational studies on caffeic-acid-derived esters and related phenolics, where antioxidant efficacy was linked to effective donor characteristics, hydroxyl configuration, and radical delocalization, rather than merely an increase in oxygenated substituents [30,31]. In this regard, the current findings endorse the perspective that methylated derivatives need not to be seen as electronically deactivated entities, but rather as structurally reconfigured species whereby donor potential may be preserved or, in certain instances, enhanced.

## 4. Materials and Methods

### 4.1. Standards

Caffeic acid was obtained from Merck (Darmstadt, Germany), whereas ferulic acid, gallic acid, and syringic acid were obtained from Sigma-Aldrich (Steinheim, Germany). Quantification was performed using external standards for calibration. Stock solutions of all reference compounds were prepared in methanol (Merck KGaA, Darmstadt, Germany). Working standard solutions were prepared by diluting the stock solutions with 63% methanol containing 2 g/L BHT (Sigma-Aldrich, Steinheim, Germany) and 7 mol/L hydrochloric acid (Merck KGaA, Darmstadt, Germany) to obtain concentrations ranging from 0.55 to 26 mg/L. Stock and working solutions were stored at −17 °C in the dark.

#### Chemical Reagents

Dichloromethane and acetic acid were obtained from Merck KGaA (Darmstadt, Germany). Propanone and methanol were of analytical grade. Glacial acetic acid and acetonitrile were HPLC grade from Merck KGaA, Darmstadt, Germany. Iron chloride and sodium acetate were acquired from Sigma-Aldrich (Steinheim, Germany).

### 4.2. Vegetal Material

**Figure 10:**
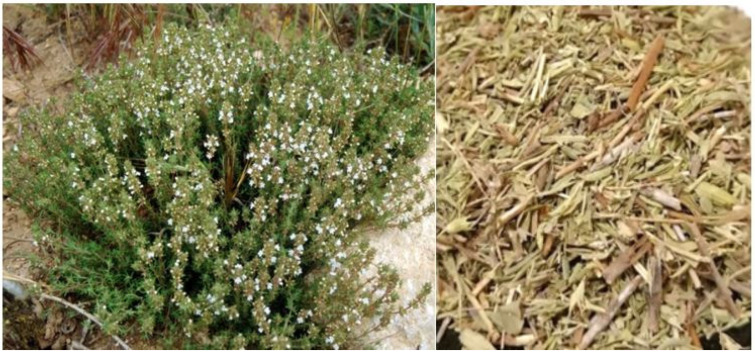
*Satureja montana*.

The botanical specimens (*Satureja montana*) were harvested during the flowering phase in Spain, specifically in Valencia, from various sections of individual plants within the same population (Figure 10). Following harvest, they were air-dried and subsequently vacuum-sealed for storage under optimal conditions, maintaining a dry, dim environment at room temperature until the time of preparation of the plant extracts.

### 4.3. Sample Processing

The methodology employed for extracting the dried samples involved the following steps: Methanol was used as the solvent at a ratio of 200 g per plant material sample. Upon complete evaporation, the extracts were subjected to liquid–liquid extraction to isolate samples enriched in phenolic compounds. Using 4.5% sodium hydroxide, the phenolic compounds in the crude methanol extract were extracted. These chemicals were acidified with 4.5% hydrochloric acid (pH~7) and then extracted with chloroform. The resulting extract, abundant in phenols, was rinsed with 4.5% NaHCO_3_, yielding Extract 1. Next, the solution was acidified with 4.5% hydrochloric acid, and the phenolic acids were extracted with chloroform to obtain Extract 2. Ultimately, the extracts were separated into two parts and thoroughly evaporated under reduced pressure.

#### 4.3.1. Modification Using Diazomethane

Diazomethane was synthesized following the procedure described by Moore and Reed (1961) [32]. The experimental procedure began by adding ethanol, water, and potassium hydroxide to the reaction flask, followed by stirring and heating to 65 °C to dissolve the KOH. The distillation setup consisted of dry ice, a receiving flask containing the acid chloride in ether, and ice baths at −78 °C and water at 65 °C. The Diazald solution in ether was immediately added to the reaction flask, quickly replacing the distillation setup.

**Figure 11:**
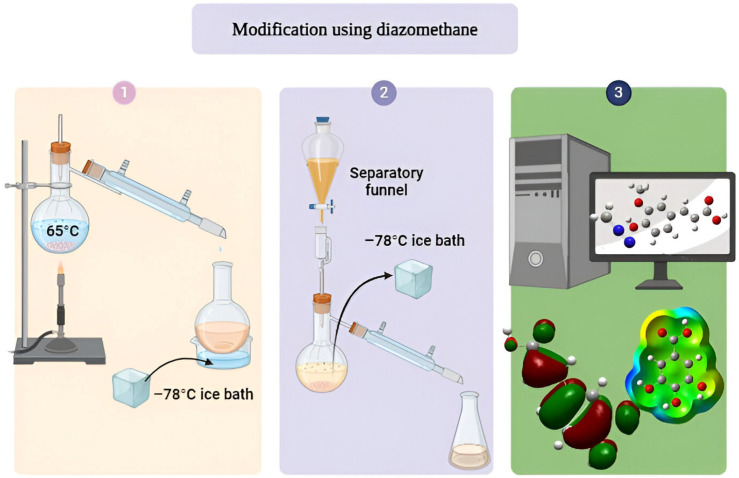
Experimental procedure for the modification of natural antioxidants with diazomethane.

Once diazomethane distillation ceased and the yellow color disappeared, the assembly was removed and cleaned. More ether was added to ensure a complete transfer of diazomethane to the receptor. For five hours, the reaction blend was stirred at room temperature. Subsequently, via low-pressure evaporation at 40 °C, the solvent was extracted until a dry residue was achieved (Figure 11).

The reactions of each pure phenolic acid with diazomethane were computationally modeled to obtain their respective methyl esters. The data are detailed in the Supplementary Materials.

#### 4.3.2. Determination of Total Phenolic Content (Folin–Ciocalteu Assay)

We used a spectrophotometer to assess the concentration of phenolic compounds in both the native and diazomethane-modified extracts using the Folin–Ciocalteu method. This approach uses phenolic compounds to reduce a phosphomolybdic-phosphotungstic reagent under alkaline conditions, resulting in the creation of a blue complex that can be detected via spectrophotometry. This study employed the test to assess the overall frequency of Folin-responsive phenolic functionalities in extracts and to investigate the compositional changes generated by diazomethane treatment. The results were reported in mmol/g and used to compare EN1, EN2, EM1, and EM2.

#### 4.3.3. Iron-Reducing Antioxidant Capacity (FRAP)

**Figure 12:**
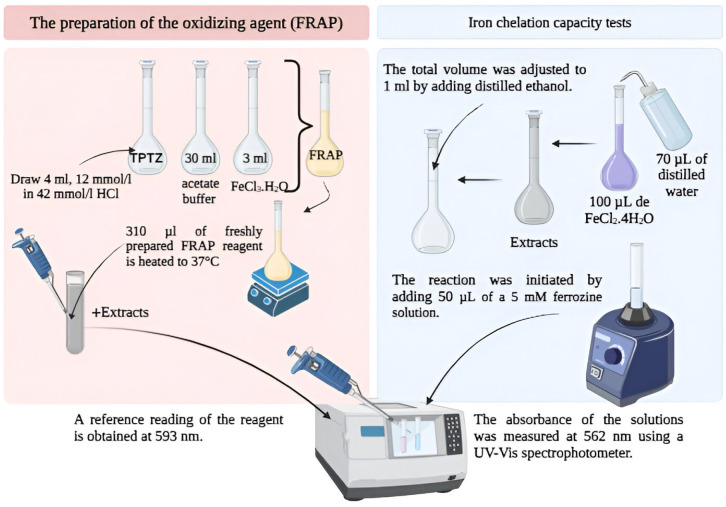
Diagram of antioxidant capacity using the FRAP and Ca–Fe method.

Originally conceived by Benzie and Strain in 1996, the FRAP technique was initially developed to evaluate the reducing capacity in plasma [33]. As research progressed, adaptations of this assay emerged to assess the antioxidant potential of botanical products, as evidenced by numerous studies [34,35,36]. The procedure measures the amount of ferrous 2,4,6-tripyridyl-triazine (TPTZ) reduced until a colored product is obtained (Figure 12). This procedure uses TPTZ, a ferric salt, as an oxidizing agent. The process encompasses the subsequent procedures: initializing the oxidizing agent (FRAP) necessitates blending TPTZ (4 mL, 12 mmol/L in 42 mmol/L hydrochloric acid), 30 mL of acetate buffer, and 3 mL of FeCl_3_·H_2_O (22 mmol/L). A baseline measurement of the freshly prepared FRAP reagent at 593 nm is taken after heating 310 μL of it to 37 °C to determine the FRAP value. Following this, 10 μL of the sample (extracted using water, 60% methanol, and ethanol) and 29 μL of water are introduced. Measurements of absorbance are conducted at 0, 5, and 35 min. Next, apply ΔA = A5 min − A0 min and ΔA = A35 min − A0 min to compute the change in absorbance. The assessment of sample reducing capacity was conducted by computation using the following formula: CR (%) = (AB − AA) × 100; where CR denotes the reducing capacity, AB represents the absorption of the control sample (taken as 100%), and AA signifies the absorption of the tested sample. The IC_50_ value was determined by charting the percentage decreases versus concentration [9].

#### 4.3.4. Metal Chelating Ability (Ca–Fe)

Iron chelation capacity tests of extracts and standard antioxidants were performed according to the procedure described in previous studies [37]. In this method, a 2 mM solution containing 100 μL of FeCl_2_·4H_2_O and 70 μL of water was added, and the solution was distilled to 50 μL, containing the samples at concentrations of 20, 35, and 45 μg/mL. Distilled ethanol was added, and the final amount was set to 1 mL. The reaction was initiated by adding 50 μL of a 5 mM ferrozine solution to the mixture. The solution was thoroughly mixed, then incubated at room temperature for 10 min. Then, using a UV-Vis spectrophotometer, the absorbance of the solutions was measured at 562 nm.

The calculation of the chelating capacity percentage was performed using the formula IC (%) = [(BC − BS)/BC] × 100, where BC and BS denote the absorbance readings at predetermined intervals for the item being examined and the reference sample, respectively. A plot illustrating the correlation between chelating capacity percentage and concentration was created by applying the linear equation, which enabled the determination of the IC_50_ value [38]. Various methodologies are reported in the literature for determining the antioxidant activity of plant extracts. However, the variability in results across trials makes direct comparison more difficult. These two methods (FRAP and Ca–Fe) were chosen based on their simplicity, sensitivity, and reproducibility capacity [39,40].

### 4.4. Computational Method

This research utilized a Density Functional Theory (DFT) methodology to examine the structural, electrical, and thermochemical characteristics of the phenolic compounds under review. The B3LYP hybrid functional was chosen due to its recognized equilibrium between precision and computational expense for antioxidant phenolic systems [41,42]. The 6-311++G(d,p) basis set was employed to furnish a characterization of electronic interactions, frontier molecular orbital energies, and bond dissociation phenomena pertinent to antioxidant activity. This technique has worked well in past investigations of phenolic antioxidants that are structurally similar [43].

**Figure 13:**
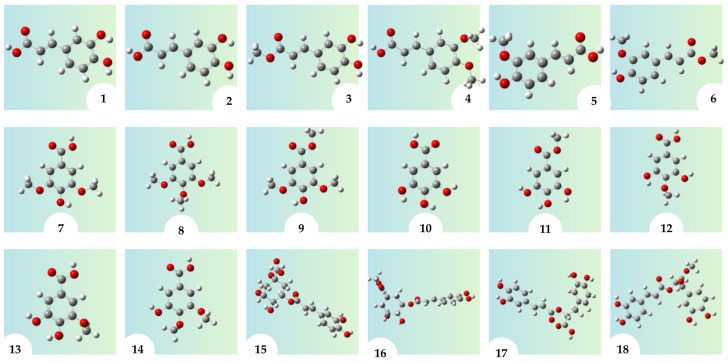
Lowest-energy conformers of compounds **1**–**18** used in the DFT calculations. The spheres represent the atoms, with gray for carbon (C), red for oxygen (O), and white for hydrogen (H).

We used Gaussian 16 software Rev. C.01 [44] to do all of the math. The starting geometries of the neutral antioxidants were derived from isomeric forms already documented in the literature as the most stable or typical structures for each chemical (Figure 13). To prevent only dependence on literature assignments, these initial structures were independently reassessed in this study by conformational analysis. Specifically, relaxed potential-energy scans followed by geometry optimizations were conducted to ascertain the lowest-energy conformer for each antioxidant. Consequently, the structures examined in this study represent the most stable conformers derived from the integrated literature-informed and computational screening methodology.

At 298.5 K, geometry optimizations were done at the B3LYP/6-311++G(d,p) level. After that, frequency computations were done to confirm the type of stationary points and to get zero-point energy and Gibbs free energy adjustments [45,46]. After that, radical species were made from the optimized neutral structures and handled using the appropriate unconstrained formalism. The optimized Cartesian coordinates of all final structures utilized in this work are included in the Supporting Information in XYZ format to assure repeatability and allow the reuse of the computational models.

#### 4.4.1. Solvation Effect Correction

Since antioxidant processes occur in environments where solvent effects can influence the thermodynamic and electronic properties of the compounds, we performed simulations in two media: water (a polar solvent) and benzene (a nonpolar solvent). To model these effects, we employed the SMD (Solvation Model Based on Density), which allowed for a more accurate assessment of solvation interactions and their impact on BDE (Bond Dissociation Energy) and IP (Ionization Potential) calculations.

In particular, for BDE calculations, a solvation correction was applied to the enthalpies of the radicals and molecules involved. Thus, BDE was defined as follows:

BDE = *Hx* + *Hy* − *Hz* (1)

where *Hx* stands for the enthalpy of the resulting radical post-hydrogen extraction reaction, *Hy* represents the enthalpy of the hydrogen atom (0.4998 Hartree), and *Hz* indicates the enthalpy of the initial molecule.

However, for solvent-phase calculations, the enthalpies of each species were adjusted using the solvation correction:

*H*_solv_ = *H*_gas-phase_ + Δ_solv_*H* (2)

where Δ_solv_*H* represents the solvation contribution specific to the medium, in particular, for the hydrogen atom, we applied the solvation correction values reported in the literature (−4.0 kJ/mol in water and −0.5 kJ/mol in benzene) to adjust its enthalpy [47]. The same correction was applied to radicals and complete molecules, ensuring that the BDE values accurately reflect the compounds’ behavior in each solvent.

Finally, the adiabatic ionization potentials (IP) [48] were computed using the equation:

IP = *E*_cation_ − *E*_molecule_ (3)

and the interactions between the hydroperoxyl radical and phenolic acids were evaluated using the hydrogen-atom-transfer (HAT) mechanism, incorporating solvent effects to provide a more realistic interpretation of antioxidant reactivity.

#### 4.4.2. Assessment of Frontier Molecular Orbitals

A thorough analysis of the frontier molecular orbitals was conducted for each polyphenol and its optimized derivatives, with the energy gap simultaneously evaluated. Electrostatic potential maps were examined to visualize the charge distributions within the molecules in three dimensions. These maps provide insights into areas of varying charges, offering valuable information about molecular interactions [49].

#### 4.4.3. Comprehensive Descriptive Parameters

A molecule’s global description provides important insights into its chemical reactivity, susceptibility to structural alterations, and responsiveness to external factors. These characteristics, which indicate how the electron density responds to changes in the external potential and the electron count [50], include hardness, softness, electronegativity, chemical potential, and the electrophilicity index. Therefore, these general descriptors are essential for comparing the characteristics of different compounds.

The system’s overall stability is reflected in its hardness [51]. Essentially, chemical hardness indicates how resistant the electronic cloud of atoms, ions, or molecules is to deformation or polarization in response to minor perturbations during chemical processes. Conversely, Chemical Softness measures a molecule’s capacity to accept electrons and is inversely related to chemical hardness, with a direct connection to the specific groups or atoms within the molecule. Within Density Functional Theory (DFT), chemical potential is defined as an electron’s tendency to stray from equilibrium. It is derived from the energy’s first derivative with respect to the number of electrons. It also denotes the ability to draw electrons into a chemical connection and is the opposite of electronegativity. The electrophilicity measure provides insights into a substance’s electrophilicity. Comprehensive reactive descriptors can be determined using two distinct methodologies. One approach is based on the overall electronic energy of the nonpartisan entity and its associated anions and cations, which may be determined from the nonpartisan entity’s structure while holding the external potential constant, or “vertical energy” [52].

## 5. Conclusions

In conclusion, *Satureja montana* extracts represent a significant source of phenolic antioxidants, whose properties are markedly modified by diazomethane derivatization. Experimentally, the native extracts exhibited the highest phenolic content and reducing capacity, with EN1 achieving 1278.54 mmol/g and a FRAP value of 9.36, while EM2 exhibited only 1.99 mmol/g and demonstrated a lower, yet still measurable, reducing capacity of 2.08. The modified extracts, on the other hand, had the strongest Fe^2+^-chelating behavior, with IC_50_ values of 0.70 mg/mL for EM1 and 0.82 mg/mL for EM2. This means that methylation does not always make antioxidant activity stronger, but it does move it around between pathways that reduce and chelate metals. The DFT results at the molecular level showed that the suggested methylation reactions are possible from a thermodynamic point of view. This is especially true for compounds **18** (−57.10 kcal/mol), **16** (−53.96), **6** (−53.34), and **3**, **9**, and **11** (−52.71). Furthermore, solvent-dependent BDE and IP values indicated that antioxidant reactivity is influenced by both molecular structure and solvent effects. Methyl 3,4-dihydroxycinnamate was the best hydrogen donor of all the species that were looked at. It had the lowest BDE (71.60 kcal/mol) and the best ΔG°r toward HOO• (−11.06 kcal/mol). In general, the experimental and theoretical results together show that diazomethane treatment changes the antioxidant profile of the extract instead of making all of its activities go up at the same time. They also show that DFT descriptors are a useful way to understand these changes.

## Figures and Tables

**Table 1:** Frontier molecular orbital energies and global reactivity descriptors of compounds **1**–**18**.

Compound	E_HOMO_ (eV)	E_LUMO_ (eV)	∆E (eV)	ղ (eV)	S (eV)	ω (eV)	µ (eV)	X (eV)	Dipole Moment (debye)	Polarizability (au)
**1**	−0.231	−0.078	0.153	0.0765	13.072	0.1560	−0.1545	0.1545	5.09	137.27
**2**	−0.229	−0.072	0.157	0.0785	12.739	0.1443	−0.1505	0.1505	3.01	166.4
**3**	−0.227	−0.073	0.154	0.077	12.987	0.1461	−0.15	0.15	4.73	151.5
**4**	−0.228	−0.076	0.152	0.076	13.158	0.1520	−0.152	0.152	5.11	167.88
**5**	−0.225	−0.076	0.149	0.0745	13.423	0.1520	−0.1505	0.1505	2.91	152.72
**6**	−0.221	−0.071	0.15	0.075	13.333	0.1421	−0.146	0.146	1.99	167.06
**7**	−0.225	−0.054	0.171	0.0855	11.696	0.1138	−0.1395	0.1395	2.81	131.76
**8**	−0.233	−0.059	0.174	0.087	11.494	0.1225	−0.146	0.146	1.48	144.92
**9**	−0.221	−0.048	0.173	0.0865	11.561	0.1046	−0.1345	0.1345	0.54	144.77
**10**	−0.239	−0.059	0.18	0.09	11.111	0.1233	−0.149	0.149	2.34	103.4
**11**	−0.235	−0.053	0.182	0.091	10.989	0.1139	−0.144	0.144	1.52	116.45
**12**	−0.24	−0.062	0.178	0.089	11.236	0.1281	−0.151	0.151	4.35	117.24
**13**	−0.24	−0.059	0.181	0.0905	11.050	0.1235	−0.1495	0.1495	1.98	117.47
**14**	−0.245	−0.063	0.182	0.091	10.989	0.1303	−0.154	0.154	1.21	130.48
**15**	−0.225	−0.073	0.152	0.076	13.158	0.1461	−0.149	0.149	3.93	245.63
**16**	−0.224	−0.072	0.152	0.076	13.158	0.1441	−0.148	0.148	4.75	257.94
**17**	−0.221	−0.076	0.145	0.0725	13.793	0.1521	−0.1485	0.1485	3.16	262.35
**18**	−0.222	−0.072	0.15	0.075	13.333	0.1441	−0.147	0.147	2.28	275.39

**Table 2:** Gibbs free energy and enthalpy changes for the reaction of selected phenolic compounds with the hydroperoxyl radical (HOO•).

Compound	∆G_ArOH_+.OOH	∆G_ArO._+HOOH	∆Gr kcal/mol	∆H_ArOH_+.OOH	∆H_ArO._+HOOH	∆Hr kcal/mol
Caffeic acid	−501,821.02	−501,831.49	−10.47	−498,581.22	−498,593.28	−12.07
3,4-Dihydroxy-5-methoxybenzoic acid	−525,117.13	−525,124.20	−7.07	−521,728.65	−521,735.10	−6.45
Ferulic acid	−526,469.67	−526,475.88	−6.21	−523,070.75	−523,077.62	−6.88
Gallic acid	−500,471.76	−500,479.78	−8.02	−497,243.02	−497,250.56	−7.54
Methyl 3,4-dihydroxycinnamate	−526,472.18	−526,483.24	−11.06	−523,072.68	−523,085.37	−12.70
Methyl Ferulate	−551,120.91	−551,127.70	−6.78	−547,562.29	−547,569.05	−6.76
Methyl Gallate	−525,123.84	−525,130.11	−6.27	−521,736.92	−521,742.53	−5.62
Syringic acid	−549,770.66	−549,775.44	−4.78	−546,225.70	−546,228.63	−2.93
Methyl Syringate	−574,419.27	−574,427.21	−7.94	−570,712.17	−570,719.98	−78.1
Chlorogenic acid	−909,029.73	−909,031.97	−2.25	−903,176.18	−903,185.98	−9.80

## Data Availability

Data are contained within the article and Supplementary Materials.

## Supplementary Materials

The following supporting information can be downloaded at: https://www.mdpi.com/article/10.3390/molecules31091375/s1.
