# Supplementary material for: Investigation of the Antioxidant Activity of Hydroxycinnamic Acids, Hydroxybenzoic Acids, and Their Synthetic Diazomethane Derivatives
Source: Molecules. 2026 Apr 22;31(9):1375. doi: 10.3390/molecules31091375 (PMC13164996; doi:10.3390/molecules31091375)
Supplement: Supplementary file 1 [file molecules-31-01375-s001.zip › molecules-4196615-supplementary.pdf]

Supporting Information

Figure S1. Initial inputs for geometry optimizations 1-18.

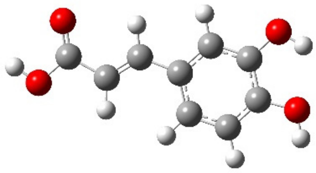	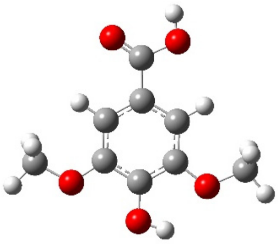	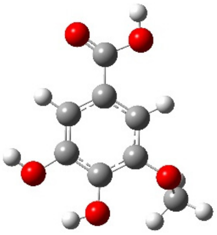
**1**	**7**	**13**
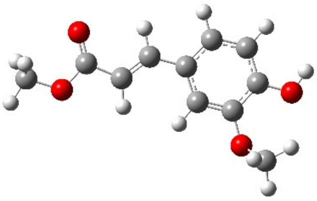	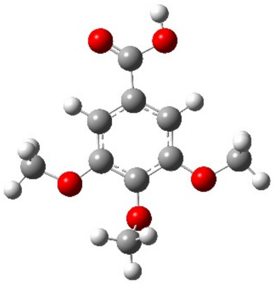	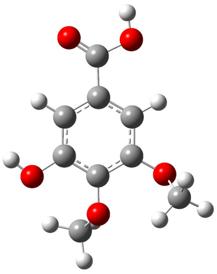
**2**	**8**	**14**
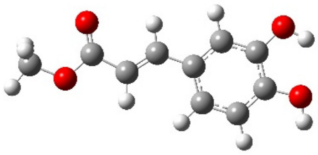	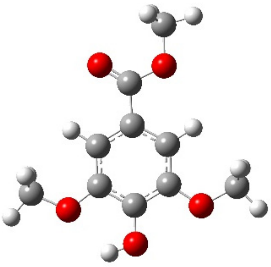	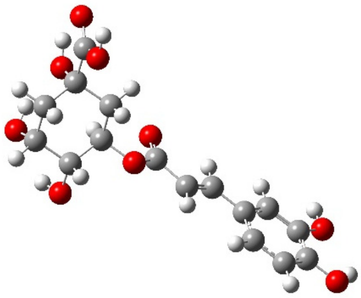
**3**	**9**	**15**
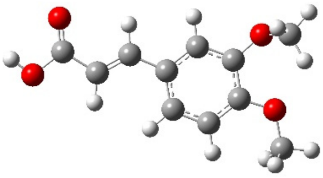	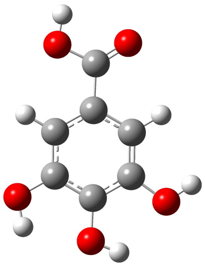	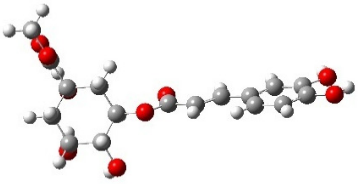
**4**	**10**	**16**

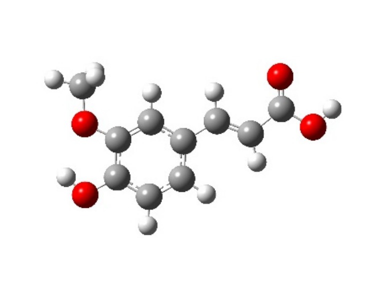	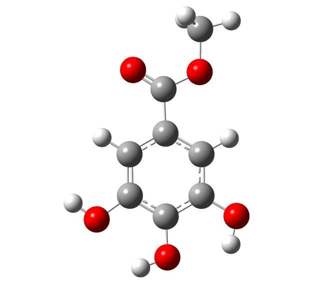	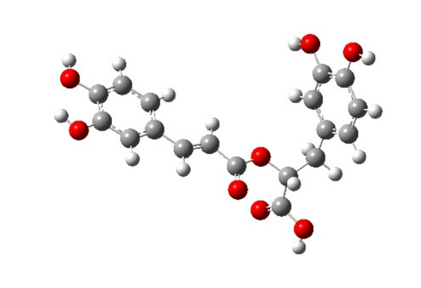
**5**	**11**	**17**
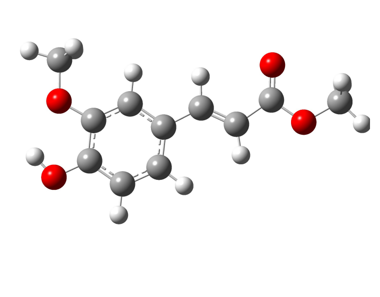	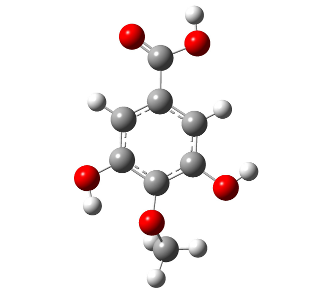	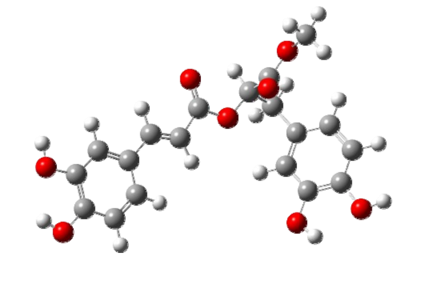
**6**	**12**	**18**

FigureS2. Reaction of phenolic acids with diazomethane in kcal/mol.

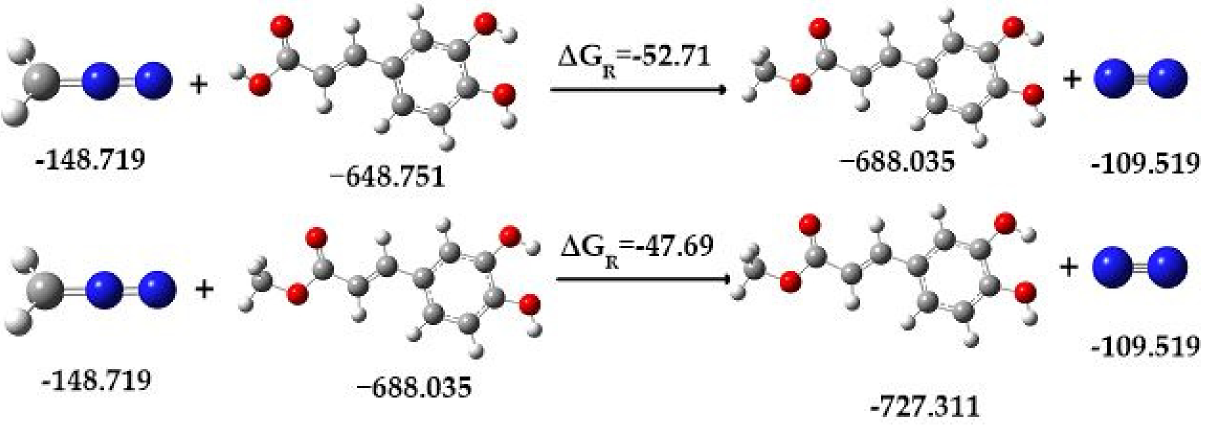

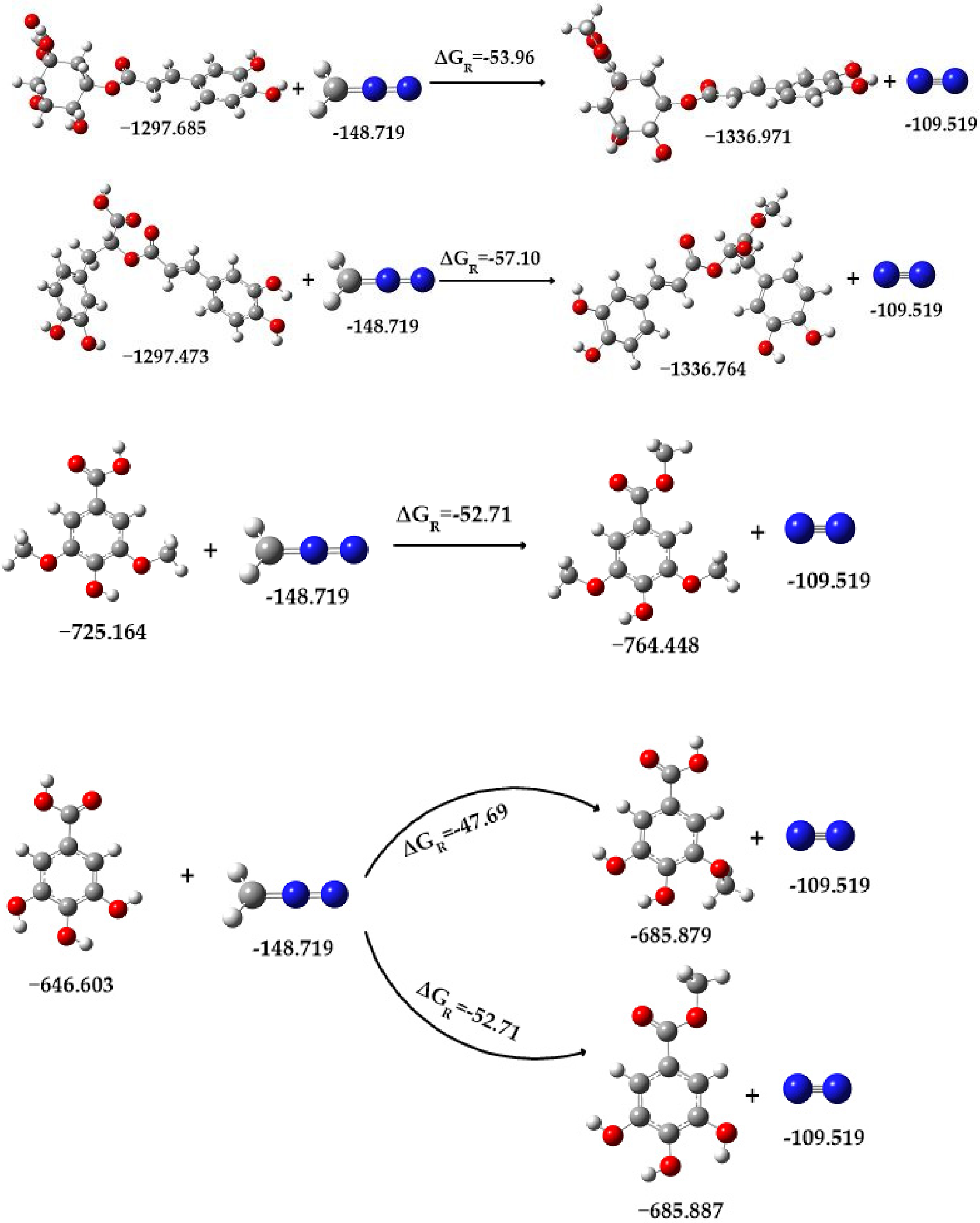
ΔGR=-53.96
−1297.685
-148.719
−1336.971
-109.519
ΔGR=-57.10
−1297.473
-148.719
−1336.764
-109.519
ΔGR=-52.71
−725.164
-148.719
−764.448
-109.519
ΔGR=-47.69
ΔGR=-52.71
−646.603
-148.719
-685.879
-109.519
−685.887
-109.519

Water

#	Compound	Energy (Hartree)	H (Hartree)	G (Hartree)	BDE (kcal/mol)	IP (kcal/mol)
**4**	2−propenoic acid	−727.26548	−727.037431	−727.096678		
	R1−2−propenoic acid	−726.596903	−726.382617	−726.443681	97.64825184	
	Cation	−727.06256	−726.833779	−726.89421		127.3329088
**8**	3.4.5−T	−764.369384	−764.142509	−764.202229		
	R1−3.4.5−Trimetoxy	−763.704716	−763.490953	−763.550785	95.60384707	
	Cation	−764.16402	−763.936602	−763.996237		128.8665261
**13**	3.4−D	−685.777581	−685.610478	−685.662874		
	R1−3.4−D	−685.142437	−684.987638	−685.04027	77.58447092	
	R2−3.4−D	−685.142477	−684.987706	−685.03992	77.54180072	
	Cation	−685.559141	−685.392043	−685.444971		137.0717553
**12**	3.5−D	−685.778545	−685.611346	−685.663556		
	R1−3.5−D	−685.134783	−684.980368	−685.033581	82.69109033	
	R2−3.5−D	−685.106669	−684.95312	−685.006619	99.78929208	
	Cation	−685.559854	−685.392822	−685.445617		137.2292586
**14**	3−H	−725.068364	−724.871714	−724.92827		
	R1−3H	−724.433852	−724.249561	−724.306223	77.15337636	
	Cation	−724.85607	−724.659083	−724.716607		133.2151219
**16**	3−O−methyl−ester	−1336.864576	−1336.477111	−1336.560789		
	R−methyl−ester	−1336.232928	−1335.857567	−1335.941304	75.51622103	
	Cation	−1336.654632	−1336.266836	−1336.350072		131.7404898
**15**	AC	−1297.562282	−1297.204828	−1297.284977		
	R1−AC	−1296.930602	−1296.585236	−1296.66571	75.54634118	
	Cation	−1297.352281	−1296.994282	−1297.074222		131.7762575
**5**	AF	−687.975246	−687.776452	−687.831158		
	R1−AF	−687.339086	−687.152845	−687.207822	78.06576572	
	Cation	−687.767928	−687.568884	−687.624946		130.092667
**10**	AG	−646.484927	−646.34764	−646.395768		
	R1−AG	−645.848456	−645.72361	−645.771736	78.33119949	
	R2−AG	−645.851576	−645.726305	−645.774054	76.6400789	
	R3−AG	−645.813161	−645.690322	−645.736929	99.21951935	
	Cation	−646.258003	−646.120966	−646.170188		142.3954908
**17**	AR	−1297.341558	−1297.001641	−1297.084423		
	R1−AR	−1296.712953	−1296.38511	−1296.467195	73.62555449	
	Cation	−1297.133945	−1296.793282	−1296.876802		130.2777803
**7**	AS	−725.080009	−724.882491	−724.937658		
	R1−AS	−724.445451	−724.260432	−724.315707	77.09439108	
	Cation	−724.866561	−724.668934	−724.725433		133.9392603
**1**	CAF	−648.674843	−648.506437	−648.557635		
	R1−CAF	−648.046253	−647.889961	−647.940902	73.59104183	
	R2−CAF	−648.035667	−647.879492	−647.930597	80.16037074	
	Cation	−648.462471	−648.293579	−648.345275		133.2640671
**3**	M−34	−687.975216	−687.777115	−687.832762		

	R1−M−34	−687.347192	−687.161201	−687.216483	73.23838514	
	R2−M−34	−687.33631	−687.150545	−687.206231	79.92505711	
	Cation	−687.764293	−687.56575	−687.622057		132.3548153
**6**	MAF	−727.27556	−727.04706	−727.105845		
	R1−MAF	−726.639995	−726.423922	−726.482854	77.77146681	
	Cation	−727.06968	−726.84088	−726.900731		129.1903176
**18**	MAR	−1336.647971	−1336.278013	−1336.365344		
	R1−MAR	−1336.016103	−1335.65822	−1335.744857	75.67246928	
	Cation	−1336.437146	−1336.066665	−1336.153697		132.29332
**2**	MF	−727.267331	−727.039431	−727.098973		
	R1−MF	−726.63739	−726.421751	−726.481979	74.34655544	
	Cation	−727.059025	−726.830474	−726.891935		130.7126399
**11**	MG	−685.785512	−685.618425	−685.670863		
	R1−MG	−685.144896	−684.990329	−685.043045	80.88262669	
	R2−MG	−685.152867	−684.997861	−685.049983	76.15627409	
	Cation	−685.56076	−685.393821	−685.448219		141.0325543
**9**	MS	−764.380319	−764.153144	−764.212931		
	R1−MS	−763.746435	−763.53171	−763.591249	76.7022017	
	Cation	−764.169024	−763.941623	−764.001896		132.5882464

Benzene

#	Energy (Hartree)	H (Hartree)	G (Hartree)	BDE (kcal/mol)	IP (kcal/mol)
**4**	-727.251144	−727.022668	−727.082209		
	−726.580267	−726.365367	−726.425464	101.342362	
	−727.018179	−726.788387	−726.847460		146.1862364
**8**	−764.355591	−764.12814	−764.188041		
	−763.68496	−763.471623	−763.531811	100.8503997	
	−764.122731	−763.89405	−763.95245		146.8921773
**13**	−685.76611	−685.598349	−685.650475		
	−685.130084	−684.974771	−685.026725	80.18107833	
	−685.132648	−684.977332	−685.029232	78.57404315	
	−685.517029	−685.348847	−685.401012		156.5632535
**12**	−685.766641	−685.598771	−685.650459		
	−685.120938	−684.966136	−685.019088	85.86437301	
	−685.095158	−684.941097	−684.994342	99.06640862	
	−685.516200	−685.348253	−685.399491		157.1524788
**14**	−725.056158	−724.858858	−724.915687		
	−724.422687	−724.237859	−724.294067	78.5627481	
	−724.814393	−724.616546	−724.672953		151.7082628
**16**	−1336.841456	−1336.453216	−1336.536565		
	−1336.211394	−1335.835325	−1335.918292	76.61246877	
	−1336.602363	−1336.213019	−1336.293425		150.0315748
**15**	−1297.539541	−1297.181138	−1297.261057		

	−1296.909425	−1296.563233	−1296.6425	76.62125381	
	−1297.300448	−1296.940827	−1297.018287		150.0315748
**5**	−687.962315	−687.762999	−687.817453		
	−687.323491	−687.136996	−687.191693	81.70277311	
	−687.723481	−687.5235	−687.577793		149.8690515
**10**	−646.474502	−646.336453	−646.384275		
	−645.839439	−645.713976	−645.761786	79.49019753	
	−645.839925	−645.714115	−645.761959	79.40297461	
	−645.802608	−645.679235	−645.725662	101.2902793	
	−646.21436	−646.077191	−646.12355		163.2398854
**17**	−1297.314263	−1296.973406	−1297.056508		
	−1296.687759	−1296.359219	−1296.441871	74.28819766	
	−1297.079517	−1296.737718	−1296.817221		147.3038192
**7**	−725.067009	−724.86885	−724.92374		
	−724.429902	−724.244523	−724.299666	80.65107808	
	−724.82435	−724.625608	−724.680256		152.2692505
**1**	−648.661457	−648.492545	−648.544084		
	−648.034754	−647.878123	−647.929079	74.43566087	
	−648.019338	−647.86293	−647.913967	83.96931394	
	−648.415683	−648.245648	−648.296847		154.2239223
**3**	−687.961403	−687.763793	−687.816967		
	−687.33537	−687.150059	−687.20297	74.0039388	
	−687.319607	−687.134746	−687.188132	83.61289224	
	−687.718507	−687.519878	−687.572202		152.4179687
**6**	−727.262202	−727.033223	−727.091864		
	−726.623978	−726.407791	−726.46662	81.3444689	
	−727.026201	−726.796508	−726.854854		148.0913355
**18**	−1336.622998	−1336.252221	−1336.339364		
	−1335.992996	−1335.63437	−1335.720511	76.58736865	
	−1336.389519	−1336.017611	−1336.10157		146.5087729
**2**	−727.25263	−727.024368	−727.084267		
	−726.625122	−726.409298	−726.46904	74.84228281	
	−727.014528	−726.785445	−726.844526		149.4097193
**11**	−685.774612	−685.606851	−685.65893		
	−685.131671	−684.976615	−685.028888	84.35899331	
	−685.140833	−684.985321	−685.037086	78.89595219	
	−685.518089	−685.350108	−685.403185		160.9689521
**9**	−764.366797	−764.13902	−764.198703		
	−763.730304	−763.5152	−763.574759	80.33293406	
	−764.127484	−763.899735	−763.956342		150.1696254

xyz coordinates

Optimized Cartesian coordinates of methyl 3,4−dihydroxycinnamate

C	0.675267	0.035035	0.132860
C	2.056049	0.180875	0.164119
C	2.632009	1.451765	0.146072
C	1.796278	2.583528	0.106902
C	0.424344	2.419721	0.073001
C	−0.165029	1.147834	0.082993
H	0.257993	−0.964354	0.141904
H	2.680104	−0.701967	0.193014
H	−0.189429	3.312816	0.027774
O	2.325455	3.839250	0.043004
O	3.962726	1.692724	0.152464
C	−1.623522	1.049593	0.043214
C	−2.360508	−0.066316	0.069592
C	−3.830587	0.035794	0.021725
H	−2.167117	1.990437	−0.012616
H	−1.942333	−1.063111	0.125411
O	−4.411624	−1.183439	0.052605
H	−5.366847	−1.042593	0.018248
O	−4.474681	1.051225	−0.037049
C	2.935319	4.286483	1.250110
H	3.292429	5.295959	1.056314
H	2.199004	4.306915	2.059384
H	3.774639	3.645303	1.524920
C	4.836116	0.580203	0.125224
H	4.670455	−0.025600	−0.770264
H	5.842702	0.990657	0.104920
H	4.712411	−0.040547	1.017586

Optimized Cartesian coordinates from 3,4,5−Trimethoxybenzoic acid

C	0.402450	1.280777	−0.129391
C	−0.968579	1.055878	−0.014283
C	−1.434945	−0.255596	0.020486
C	−0.571053	−1.344370	−0.052669
C	0.796372	−1.114725	−0.171676
C	1.286087	0.197855	−0.221938
H	−1.672924	1.871597	0.051755
H	−0.990597	−2.339909	−0.017337
C	−2.885852	−0.550774	0.145555
O	−3.362891	−1.653975	0.190105
O	−3.648711	0.560285	0.207687
H	−4.565448	0.266323	0.286353
O	1.735988	−2.086100	−0.244214
O	0.978968	2.504251	−0.156207
O	2.612288	0.426278	−0.396914
C	0.124082	3.628790	−0.106238
H	−0.566590	3.636345	−0.954696
H	−0.444805	3.652685	0.828285
H	0.773067	4.499636	−0.158846
C	1.286113	−3.427516	−0.247054
H	2.179140	−4.041772	−0.333270
H	0.762077	−3.670225	0.682106
H	0.626784	−3.618218	−1.098648
C	3.367936	0.359257	0.808528
H	3.025053	1.123010	1.512369
H	3.283813	−0.633695	1.257436
H	4.403267	0.552208	0.534747

Optimized Cartesian coordinates from 3,4−Dihydroxy−5−methoxybenzoic acid

C	−0.244340	0.329820	0.107846
C	1.127607	0.403209	−0.020000
C	1.782772	1.639614	−0.064888
C	1.033833	2.817139	0.005456
C	−0.348025	2.748664	0.135762
C	−0.982632	1.511570	0.191180
H	−0.759755	−0.623615	0.148245
H	−0.910465	3.670363	0.202495
O	1.649843	4.032345	0.004690
O	3.128661	1.699997	−0.163382
O	1.962506	−0.678995	−0.098835
C	−2.452985	1.388062	0.331958
O	−3.052493	0.345282	0.377281
O	−3.077962	2.579111	0.405915
H	1.466640	−1.496085	−0.002100
H	−4.022220	2.396874	0.496441
H	3.474130	0.798536	−0.168572

Optimized Cartesian coordinates from 3,5−Dihydroxy−4−methoxybenzoic acid

C	−0.209979	0.336564	0.060715
C	1.174955	0.366090	−0.025694
C	1.863000	1.583441	−0.002830
C	1.156907	2.779892	0.136668
C	−0.229629	2.760014	0.213877
C	−0.899867	1.537671	0.174654
H	−0.741432	−0.605597	0.037493
H	−0.786402	3.683945	0.315041
O	1.887077	3.927709	0.194702
O	3.226986	1.517453	−0.056739
O	1.870128	−0.787285	−0.132491
C	−2.383382	1.474074	0.257231
O	−3.031219	0.463142	0.232353
O	−2.955859	2.691672	0.365486
H	−3.910090	2.549675	0.414849
H	2.809653	−0.564288	−0.100667
H	1.299609	4.675612	0.334138

C	2.276420	4.382664	−1.227429
H	2.694338	5.376756	−1.083199
H	3.073133	3.680053	−1.476031
H	1.534956	4.409124	−2.031522

C	3.828016	2.154263	−1.189023
H	3.670251	3.231365	−1.151031
H	4.891102	1.930500	−1.131337
H	3.411411	1.744911	−2.113410

Optimized Cartesian coordinates of 2-hydroxy-3,4-dimethoxybenzoic acid

C	-0.427476	-0.046827	0.015555
C	0.957294	-0.000950	0.052542
C	1.621064	1.233921	0.027950
C	0.874947	2.415346	-0.025300
C	-0.514481	2.372843	-0.056978
C	-1.156705	1.139555	-0.040828
H	-0.958447	-0.992798	0.032013
H	-1.071443	3.298663	-0.107958
C	-2.637969	1.027190	-0.081542
O	1.739080	-1.112243	0.103825
O	2.978556	1.294976	0.083734
O	1.511531	3.619383	-0.093737
O	-3.246499	-0.010272	-0.069619
O	-3.254966	2.222637	-0.134240
H	-4.205091	2.049839	-0.159434
H	1.183057	-1.896138	0.124037
C	2.148496	4.014318	1.120374
H	2.612338	4.978458	0.921998
H	1.404690	4.121896	1.915573
H	2.908346	3.288018	1.413240
C	3.623678	0.950709	-1.143124
H	4.692530	1.042545	-0.962244
H	3.384102	-0.075867	-1.426910
H	3.319915	1.644584	-1.931419

Optimized Cartesian coordinates of methyl chlorogenate

C	-1.572265	-0.170581	0.189864
C	-0.234231	-0.450012	-0.014391
C	0.725749	0.570121	0.037073
C	0.297142	1.875455	0.298661
C	-1.044833	2.156100	0.504180
C	-1.988979	1.138252	0.452128
H	0.076430	-1.470978	-0.217231
H	1.015398	2.684448	0.339448
H	-1.386995	3.163607	0.705184
O	-2.576851	-1.100868	0.158916
O	-3.296667	1.416359	0.652498
H	-2.228721	-1.970970	-0.051343
H	-3.801876	0.598480	0.571719
C	2.127659	0.217195	-0.185250
C	3.183983	1.035931	-0.152603
C	4.531994	0.488502	-0.407374
H	2.333129	-0.829094	-0.402162
H	3.120402	2.097124	0.050598
O	4.789388	-0.658139	-0.677803
O	5.466622	1.450493	-0.290835
C	6.821693	1.074388	-0.555860
C	7.558507	2.352993	-0.943368
C	7.421450	0.425098	0.688935
C	9.062662	2.090879	-1.114408
C	8.908830	0.118912	0.458654
H	7.316245	1.105750	1.539170
C	9.668405	1.403858	0.108426
H	10.717457	1.168098	-0.088704
C	9.480902	-0.544785	1.705273
H	9.622077	2.083633	0.962631
H	6.883005	-0.500015	0.908517
O	7.036705	2.911906	-2.122825
H	7.458567	2.444069	-2.855012
H	7.407007	3.089378	-0.146194
H	6.834016	0.365111	-1.387363
H	9.547340	3.059386	-1.260500
O	9.280461	1.371032	-2.319316
H	9.244113	0.425215	-2.123999
O	9.529959	0.272299	2.751469
O	9.822948	-1.698972	1.728266
O	9.048981	-0.791591	-0.619863
H	9.261765	-1.662920	-0.258514
C	10.018496	-0.308908	3.968096
H	11.036888	-0.669424	3.826503
H	9.988753	0.487674	4.705578

H 9.379378 -1.139153 4.267199

Optimized Cartesian coordinates of chlorogenic acid

C -1.592633 -0.164147 0.167516
C -0.254200 -0.444407 -0.032606
C 0.705398 0.576347 0.014851
C 0.275943 1.883304 0.266812
C -1.066512 2.164864 0.467667
C -2.010200 1.146319 0.420520
H 0.057172 -1.466508 -0.228492
H 0.993811 2.692852 0.303256
H -1.409456 3.173565 0.661169
O -2.597007 -1.094640 0.140248
O -3.318199 1.425334 0.616467
H -2.247417 -1.967570 -0.055347
H -3.823060 0.606678 0.541423
C 2.107777 0.222373 -0.201590
C 3.164896 1.040036 -0.162948
C 4.512954 0.491213 -0.411626
H 2.313191 -0.823971 -0.418299
H 3.101723 2.100831 0.042324
O 4.770906 -0.654696 -0.684838
O 5.449433 1.450982 -0.285372
C 6.804355 1.071449 -0.542710
C 7.551327 2.349777 -0.911239
C 7.389925 0.407159 0.701379
C 9.055210 2.080563 -1.073836
C 8.877892 0.094322 0.478414
H 7.281267 1.080656 1.556906
C 9.648515 1.378053 0.146515
H 10.697313 1.137426 -0.045375
C 9.432359 -0.579910 1.724221
H 9.600449 2.050816 1.006187
H 6.844261 -0.516610 0.908258
O 7.041795 2.923944 -2.088344
H 7.466223 2.462073 -2.822809
H 7.398115 3.078588 -0.107470
H 6.820686 0.369894 -1.380705
H 9.546782 3.047604 -1.205335
O 9.278648 1.373037 -2.284712
H 9.234146 0.425215 -2.102249
O 9.483674 0.233682 2.783216
O 9.762091 -1.734738 1.753913
H 9.813739 -0.275883 3.536432
O 9.024313 -0.805804 -0.606612
H 9.196945 -1.689907 -0.256163

Optimized Cartesian coordinates of (S)-rosmarinic acid

O 1.180070 1.249229 -0.406099
O 3.312480 4.132136 0.096688
O 2.982203 -3.704197 -1.198660
O 4.393403 -4.141950 1.051072
O 1.478973 3.878008 -1.246852
O 0.098948 2.281230 1.340329
O -7.026247 0.124604 1.360201
O -7.225249 -1.667245 -0.620010
C 3.571537 1.224819 -0.771901
C 2.414300 1.945907 -0.051118
C 3.801318 -0.188056 -0.275716
C 3.278564 -1.287636 -0.975271
C 4.539260 -0.428236 0.891416
C 2.309471 3.397166 -0.477684
C 3.481873 -2.592264 -0.526623
C 4.743121 -1.735294 1.347237
C 4.218800 -2.821485 0.646745
C 0.056037 1.500153 0.373652
C -3.529158 0.151385 0.154897
C -1.089065 0.737104 -0.099739
C -2.293025 0.843432 0.504933
C -4.690698 0.445619 0.904621
C -3.620027 -0.792741 -0.890636
C -5.894844 -0.177423 0.615789
C -4.831306 -1.420164 -1.178678
C -5.965339 -1.111876 -0.428322
H 3.359900 1.225424 -1.842095
H 4.470675 1.822421 -0.615013
H 2.551929 1.913746 1.027993
H 2.707361 -1.128051 -1.881845
H 4.961730 0.399893 1.445330
H 5.317583 -1.914388 2.248175
H -0.916495 0.096939 -0.952377
H -2.366484 1.521721 1.349503
H -4.653989 1.162411 1.713464
H -2.749558 -1.043525 -1.479183
H -4.896864 -2.145145 -1.979848
H 3.293038 5.068853 -0.194248
H 2.488269 -3.439550 -1.997321
H 4.918439 -4.191290 1.871863
H -7.790651 -0.391969 1.038906
H -7.256430 -2.314477 -1.348281

Optimized Cartesian coordinates of 4-hydroxy-3,5-dimethoxybenzoic acid (syringic acid)

C 0.949204 1.097690 -0.000160
C -0.431203 1.254879 -0.000114
C -1.237547 0.102285 -0.000089

Optimized Cartesian coordinates of (E)-3-(3,4-dihydroxyphenyl)prop-2-enoic acid (caffeic acid)

C -0.533367 -1.299735 -0.000109
C -1.896030 -1.593761 -0.000090
C -2.826861 -0.555731 -0.000010

<table>
<tr>
<td>C -0.658866 -1.178880 -0.000137
C 0.728401 -1.323837 -0.000183
C 1.538360 -0.177163 -0.000190
H -0.883551 2.232582 -0.000092
H -1.305807 -2.041482 -0.000145
C -2.702701 0.194697 -0.000052
O -3.487466 -0.768702 -0.000036
O -3.172089 1.490722 -0.000009
H -4.151430 1.504095 0.000021
O 1.394871 -2.537259 -0.000286
O 1.892549 2.122385 -0.000248
O 2.911136 -0.295908 -0.000229
C 1.438343 3.514321 0.000602
H 0.852881 3.727543 -0.893985
H 0.852917 3.726480 0.895465
H 2.345998 4.108530 0.000929
C 0.604158 -3.768459 0.000633
H 1.335926 -4.569861 0.000933
H -0.017300 -3.834829 0.894703
H -0.017724 -3.835909 -0.893059
H 3.307188 0.599753 -0.000174</td>
<td>C -2.401661 0.781117 0.000032
C -1.046752 1.074901 0.000006
C -0.085324 0.038974 -0.000053
H 0.176319 -2.114315 -0.000186
H -2.235495 -2.621468 -0.000147
H -0.737586 2.111030 0.000036
O -3.332819 1.810095 0.000092
O -4.207222 -0.721195 0.000013
H -4.485447 -1.655481 0.000070
H -4.239350 1.445893 0.000129
C 1.326077 0.410730 -0.000054
C 2.395578 -0.414894 0.000128
C 3.751762 0.114796 0.000104
O 4.694548 -0.896036 0.000126
H 5.600795 -0.523951 -0.000009
H 2.303334 -1.491046 0.000310
H 1.524007 1.478298 -0.000205
O 4.094606 1.311219 -0.000196</td>
</tr>
<tr>
<td>Optimized Cartesian coordinates of methyl (E)-3-(3,4-dihydroxyphenyl)prop-2-enoate (methyl caffeate)

C 0.999237 -1.283847 0.000077
C 2.355604 -1.607817 0.000045
C 3.309203 -0.590968 -0.000012
C 2.913091 0.754540 -0.000028
C 1.564786 1.078067 0.000007
C 0.580383 0.064062 0.000055
H 0.272226 -2.083185 0.000128
H 2.672061 -2.642943 0.000068
H 1.278489 2.120823 -0.000006
O 3.866624 1.763224 -0.000079
O 4.686371 -0.786602 -0.000046
H 4.943265 -1.726857 -0.000069
H 4.764796 1.378934 -0.000102
C -0.823769 0.467949 0.000078
C -1.911438 -0.331850 0.000022
C -3.260446 0.227727 0.000061
O -4.211419 -0.769066 -0.000070
H -1.844290 -1.410088 -0.000058
H -0.997373 1.539769 0.000144
O -3.567942 1.434683 0.000087
C -5.625495 -0.357829 -0.000116
H -6.185597 -1.286307 -0.000218
H -5.849840 0.225797 0.890646
H -5.849741 0.225936 -0.890811</td>
<td>Optimized Cartesian coordinates of methyl (E)-3-(4-hydroxy-3-methoxyphenyl)prop-2-enoate (methyl ferulate)

C 2.960753 -1.105094 0.000001
C 1.920494 -2.030744 0.000007
C 0.597380 -1.590263 0.000010
C 0.294772 -0.212903 0.000007
C 1.363820 0.717720 0.000001
C 2.677752 0.274091 -0.000002
H 2.158828 -3.084951 0.000010
H -0.196422 -2.323495 0.000015
H 1.141797 1.775076 -0.000001
O 3.820220 1.070666 -0.000008
O 4.273960 -1.535671 -0.000002
C -1.067553 0.310988 0.000009
H -1.145890 1.394163 0.000019
C -2.223312 -0.388809 0.000001
H -2.252489 -1.468922 -0.000012
C -3.515943 0.289031 0.000006
O -3.714820 1.518949 0.000028
O -4.553386 -0.618344 -0.000028
C 3.678292 2.527687 -0.000010
H 3.152662 2.862744 0.894539
H 4.692841 2.911887 -0.000013
H 3.152658 2.862741 -0.894559
H 4.873535 -0.762337 -0.000006
C -5.924482 -0.081200 -0.000010
H -6.095668 0.520167 -0.890802
H -6.565994 -0.955505 0.000265
H -6.095486 0.520609 0.890516</td>
</tr>
</table>

**Optimized Cartesian coordinates of methyl 3,4,5-trihydroxybenzoate (methyl gallate)**	**Optimized Cartesian coordinates of methyl rosmarinate**
C -0.196048 -1.328049 -0.000025<br>C -1.570873 -1.134796 0.000001<br>C -2.105230 0.155687 0.000008<br>C -1.259755 1.266570 -0.000009<br>C 0.120545 1.094163 -0.000034<br>C 0.653138 -0.205508 -0.000043<br>H 0.225610 -2.323159 -0.000032<br>H 0.763799 1.959089 -0.000047<br>O -1.794195 2.546161 -0.000003<br>O -3.469255 0.394947 0.000032<br>O -2.530444 -2.144881 0.000012<br>C 2.110253 -0.442469 -0.000070<br>O 2.645756 -1.564276 0.000009<br>O 2.842262 0.715621 -0.000012<br>H -2.770530 2.513586 0.000029<br>H -2.144725 -3.039869 0.000073<br>H -3.976735 -0.439210 0.000062<br>C 4.312184 0.594158 0.000065<br>H 4.673289 1.616212 0.000131<br>H 4.645461 0.067077 0.891414<br>H 4.645558 0.067157 -0.891294	O -0.839410 -0.890800 -0.869437<br>O -3.423820 -3.163005 0.253230<br>O -2.557124 3.852618 -0.232011<br>O -2.042662 -1.792545 1.464498<br>O -4.817836 3.796639 1.196062<br>O 0.406867 -2.795078 -0.537840<br>O 7.560020 -0.678980 0.633901<br>O 7.463956 1.971029 0.341589<br>C -3.123005 -0.839020 -1.687295<br>C -2.050101 -1.702477 -0.992281<br>C -3.599335 0.372734 -0.910469<br>C -2.858362 1.568415 -0.923232<br>C -4.793412 0.326919 -0.174352<br>C -2.470304 -2.195589 0.381818<br>C -3.298098 2.676510 -0.209806<br>C -5.238908 1.443553 0.547368<br>C -4.488862 2.612938 0.528167<br>C 0.356307 -1.555199 -0.621758<br>C 3.919915 -0.225767 -0.117063<br>C 1.459494 -0.613480 -0.503249<br>C 2.719070 -1.040664 -0.261435<br>C 5.139060 -0.878991 0.183039<br>C 3.925996 1.178865 -0.261467<br>C 6.304927 -0.146211 0.334849<br>C 5.103158 1.907696 -0.109482<br>C 6.295564 1.249689 0.189644<br>C -3.987927 -3.745737 1.490381<br>H -2.699909 -0.535543 -2.645981<br>H -3.966176 -1.496529 -1.901642<br>H -1.833107 -2.571145 -1.611771<br>H -1.937550 1.643460 -1.482864<br>H -5.388444 -0.576530 -0.166969<br>H -6.162257 1.401007 1.111337<br>H 1.207894 0.431140 -0.613445<br>H 2.868814 -2.110962 -0.156731<br>H 5.158854 -1.955862 0.296817<br>H 3.012031 1.705248 -0.495883<br>H -2.997355 4.533765 0.312652<br>H 5.114181 2.982558 -0.220319<br>H -5.648677 3.731234 1.700350<br>H -4.463746 -2.969137 2.084510<br>H -3.200628 -4.230310 2.063208<br>H -4.716841 -4.470037 1.146389<br>H 7.553301 -1.648330 0.732507<br>H 8.208606 1.371841 0.547620
**Optimized Cartesian coordinates of methyl 4-hydroxy-3,5-dimethoxybenzoate (methyl syringate)**<br><br>C 0.826493 1.423816 -0.000024	**Optimized Cartesian coordinates of methyl (E)-3-(4-hydroxy-3-methoxyphenyl)prop-2-enoate (methyl ferulate)**

C	-0.506898	1.008131	0.000027	C	-0.941341	0.458191	-0.149870
C	-0.818315	-0.362287	0.000025	C	-2.327436	0.505482	-0.205204
C	0.203308	-1.328246	-0.000035	C	-3.070329	-0.689927	-0.104225
C	1.525028	-0.903112	-0.000103	C	-2.406779	-1.907046	0.066103
C	1.849259	0.463165	-0.000102	C	-1.014798	-1.945245	0.119647
H	-1.305524	1.730498	0.000070	C	-0.252512	-0.765135	0.005417
H	-0.053810	-2.375585	-0.000031	H	-0.407536	1.393817	-0.236589
C	-2.214173	-0.834797	0.000050	H	-0.511775	-2.894584	0.245958
O	-2.560178	-2.029699	0.000026	C	1.202773	-0.858677	0.060751
O	-3.130427	0.186974	-0.000018	C	2.096114	0.149005	-0.041266
O	2.651220	-1.723147	-0.000213	C	3.532523	-0.101858	0.032376
O	1.237575	2.746898	-0.000005	O	4.248249	1.068544	-0.095193
O	3.172178	0.853930	-0.000172	H	1.799538	1.178492	-0.181639
H	3.737908	0.054752	-0.000184	H	1.596097	-1.861251	0.199026
C	0.217958	3.795970	0.000215	O	4.091233	-1.204212	0.188408
H	-0.404928	3.738529	-0.893648	C	5.716929	0.975230	-0.041339
H	-0.404773	3.738305	0.894171	H	6.064900	1.996834	-0.146200
H	0.775939	4.726745	0.000282	H	6.034884	0.556908	0.911608
C	2.476689	-3.176699	0.000306	H	6.086893	0.358008	-0.857839
H	3.482466	-3.583320	0.000334	H	-2.982200	-2.821161	0.144498
H	1.943731	-3.499066	0.895044	O	-4.449812	-0.596529	-0.196195
H	1.943541	-3.499694	-0.894092	O	-2.934963	1.741661	-0.434997
C	-4.558213	-0.180135	-0.000107	H	-4.880670	-1.468760	-0.113625
H	-4.798595	-0.755975	0.891112	C	-3.886740	2.238749	0.576775
H	-5.087199	0.766156	-0.000053	H	-3.414497	2.261750	1.560413
H	-4.798512	-0.755823	-0.891449	H	-4.132062	3.248959	0.262252
				H	-4.781654	1.622654	0.600954
